# A rare case report of graft-versus-host disease–related cutaneous horns of the lower lip

**DOI:** 10.1016/j.jdcr.2021.12.035

**Published:** 2022-01-08

**Authors:** Michaël Verquin, Christophe Politis, François Thonnart, Sabine Fransis, Serge Schepers

**Affiliations:** a Department of Oral and Maxillofacial Surgery, Hospital of Oost-Limburg, Schiepse Bos 6, Genk, Belgium; b Department of Pathology, Hospital of Oost-Limburg, Schiepse Bos 6, Genk, Belgium

**Keywords:** cutaneous horns, cornu cutaneum, squamous cell carcinoma, graft-versus-host disease, GVHD, graft-versus-host disease, HSCT, hematopoietic stem cell transplant

## Introduction

Cutaneous horns are hard, dense, hyperkeratotic skin lesions. They can often be found in association with viral warts, actinic keratosis, and squamous cell carcinoma, and histopathological diagnosis can be challenging. We present an atypical presentation of cutaneous horns on the lower lip, associated with oral graft-versus-host disease (GVHD) and underlying malignancy.

## Case description

**Fig 1:**
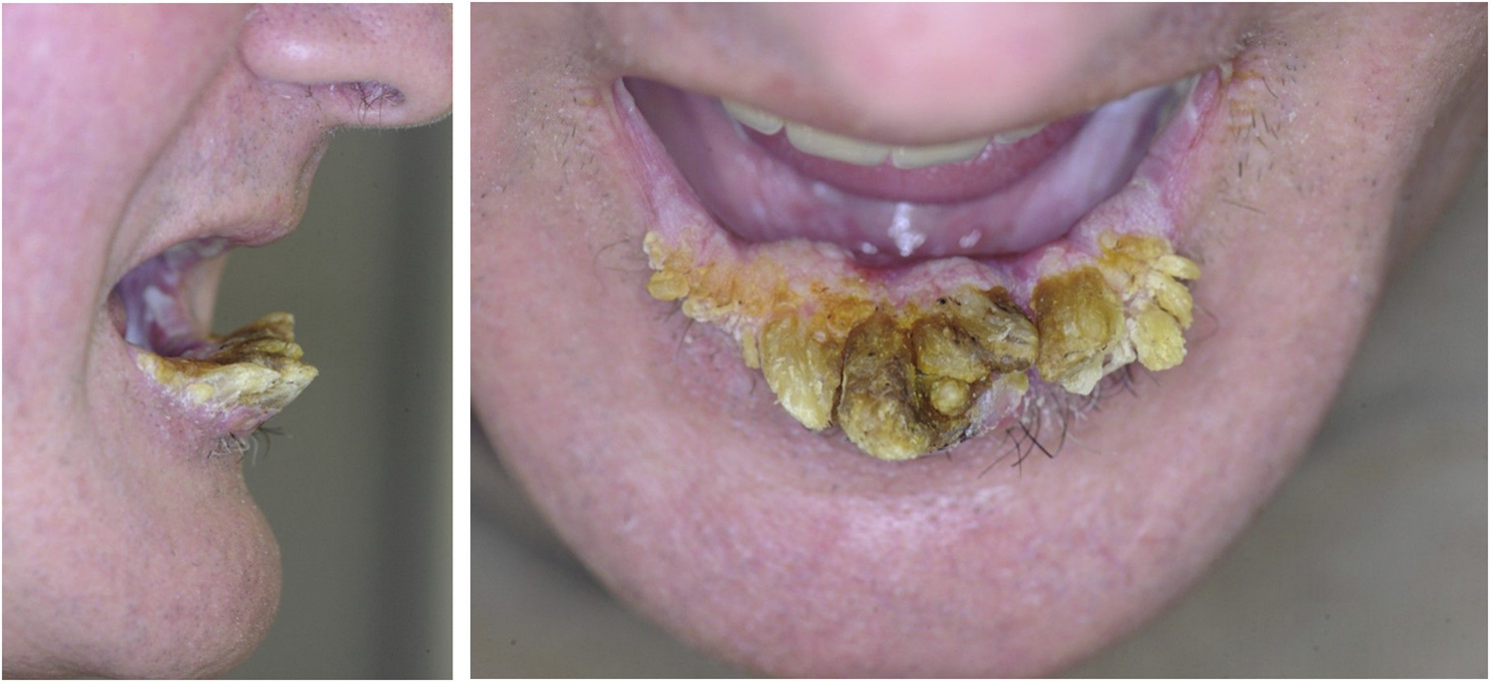
Cutaneous horns of the lower lip.

A 61-year-old white man who was active in the automobile industry presented in September 2020 with painful “tooth-like” lesions on the lower lip (Fig 1). The patient stated that the lesions had been growing for at least 3 years and that, during this time, 2 lip shaves had been attempted elsewhere in March 2017 and June 2020 with fast recurrence of the horns. No diagnosis could be made based on the pathologic assessment of the surgical specimens. Other than descriptive findings of reactive changes, hyperkeratosis, and acanthosis, there was no evidence of malignancy or oral GVHD. An abnormal, lichenoid aspect of the oral mucosa with white plaques had been present since 2010, with these plaques first arising during a severe manifestation of GVHD.

The patient was diagnosed with stage IVb follicular lymphoma (non-Hodgkin’s lymphoma) in 2001. Partial remission was achieved after 6 cycles of CHOP (cyclophosphamide, hydroxydaunorubicin, oncovin (vincristine), prednisolone). High-dose cyclophosphamide and 2 cycles of DHAP (dexamethasone, high-dose ara-C, platinol) were subsequently administered because of insufficient response, after which interferon α was prescribed as maintenance therapy. In 2005 and 2008, 4 cycles of rituximab were given because of progressive disease on control positron emission tomography-computed tomography imaging, followed by Zevalin (Y-90-Zevalin) in 2009.

Further disease progression led to a nonmyeloablative, allogeneic hematopoietic stem cell transplant (HSCT) in July 2009. Initially, cyclosporin 100 mg/day and acyclovir 800 mg/day were administered in the context of immunosuppression. After 3 months, cyclosporine was replaced by methylprednisolone 4 mg/day as maintenance therapy. Severe classic chronic GVHD was diagnosed in 2010 based on the timing of onset and clinical findings of skin sclerosis, genital phimosis, gastrointestinal symptoms, and oral lesions characterized by hyperkeratosis. Other severe post-transplant complications, including deep venous thrombosis, pulmonary embolism, epileptic insults, and liver dysfunction occurred, and 4 cycles of rituximab were initiated in response to steroid-refractory chronic GVHD.

**Fig 2:**
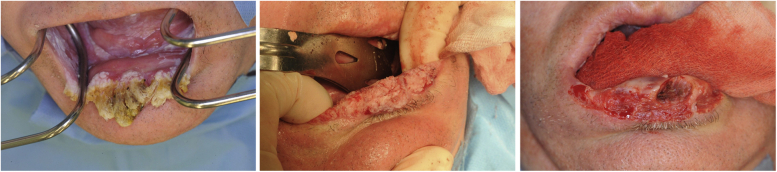
Excision of the horn-like structures.

The patient’s oral lesions never responded to therapy, and leukoplakia remained present henceforth. There is no history of similar pre-existent lesions intraoral or on the rest of the patient’s body or of prior trauma or exposure to damaging/caustic substances. The patient had no history of smoking or alcohol abuse. Medication at the time of presentation comprised acyclovir, fluconazole, methylprednisolone, and aspirin. Systemic examination revealed no abnormalities. Clinically, firm tooth-like projections were observed on the lower lip with surrounding induration and erythema spreading into the adjacent skin. There was no ulceration or discharge, and no regional lymph nodes were enlarged or painful. The patient underwent an excision of the horn-like structures with a 5-mm margin, and deep incisional biopsies of the lip and the buccal mucosa were taken (Fig 2). After excision, hemostasis was performed with monopolar cautery and dioxide laser.

**Fig 3:**
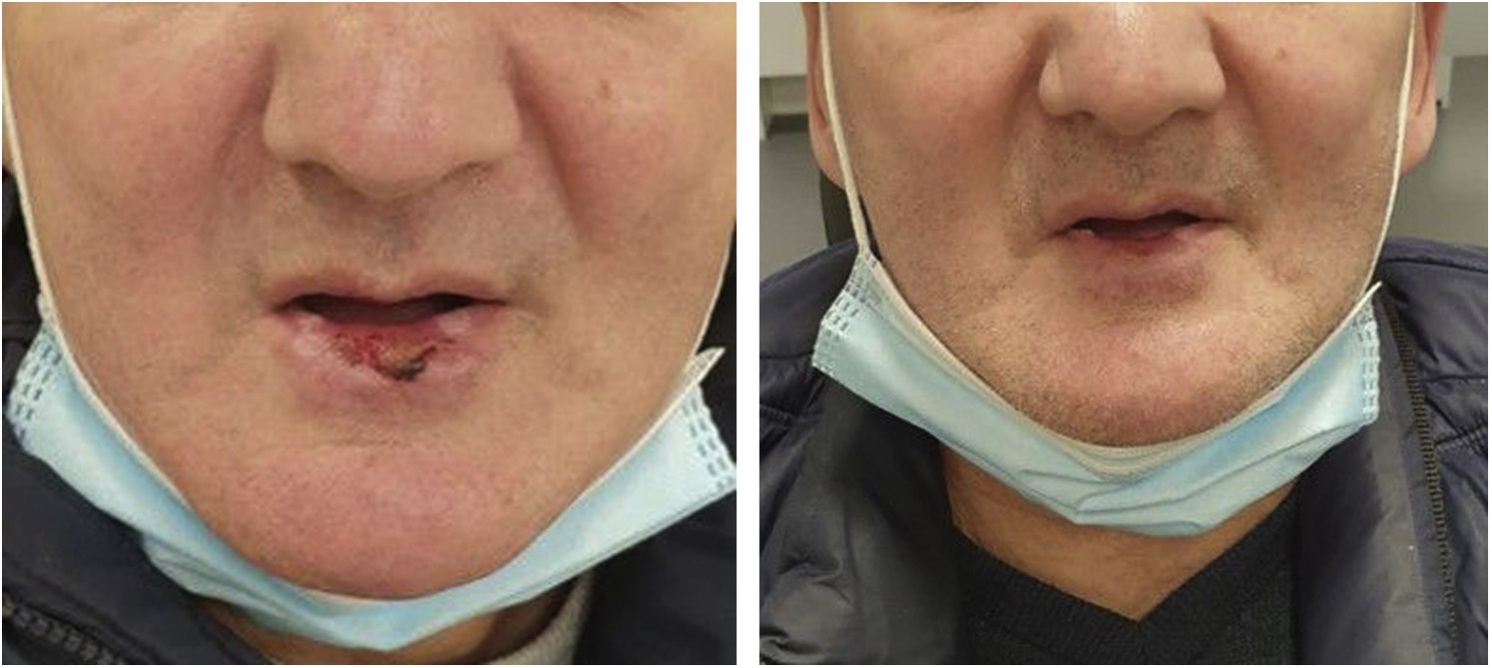
Postoperative recovery after 3 and 6 months, respectively.

The patient’s postoperative recovery period was uneventful, with complete resolution of the aesthetically displeasing cutaneous horns and underlying induration 6 months after surgery (Fig 3).

**Fig 4:**
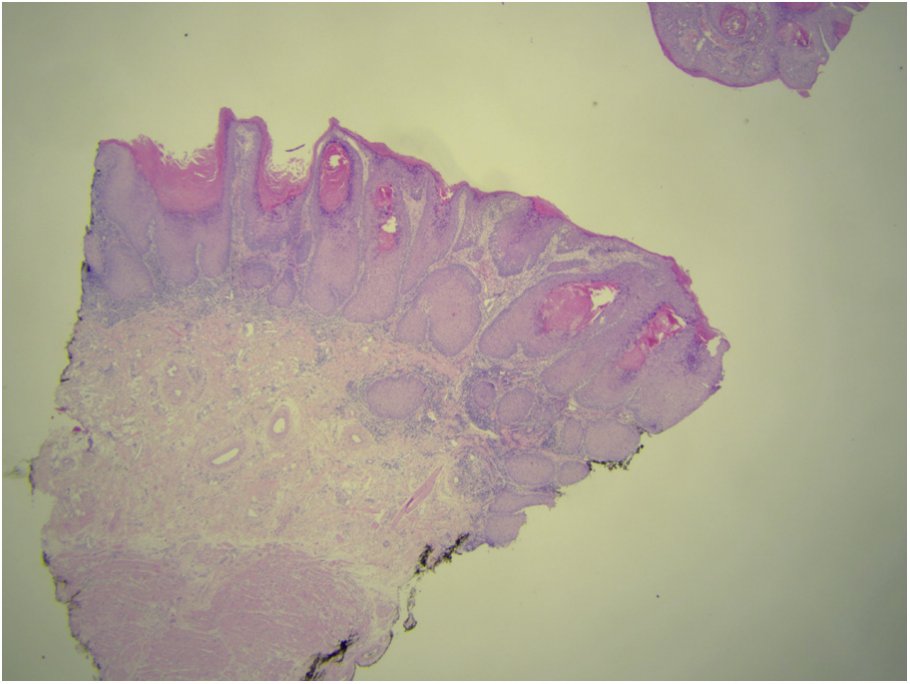
Photomicrograph showing an epithelial lesion with hyperkeratosis and acanthosis with right-side invasive growth of the squamous cell carcinoma. (Hematoxylin-eosin stain; original magnifications: ×1.25.)

The histopathological report showed a well-differentiated, invasive squamous cell carcinoma (Fig 4) with aberrant p53 expression and positive sectional margins, after verification in a tertiary hospital because of the complex nature of the findings. Due to the extent of the lesions, adjuvant radiotherapy (brachytherapy) was recommended. The patient, however, decided against further interventions because of the effective local control and improved aesthetic appearance of his lower face and was followed up regularly every 3 months. There have been no further complaints and no signs of local recurrence to date.

## Discussion

Cutaneous horns are known to have different appearances, etiology, and histopathologic features. Data on the incidence or prevalence of cutaneous horns are not available. Although cutaneous horns may occur on any site of the body, approximately 30% are found on the upper face and scalp.^1^ They may be associated with squamous cell carcinoma, viral warts, actinic keratosis, keratoacanthoma, Bowen’s disease, seborrheic keratosis, basal cell carcinoma, and Kaposi’s sarcoma, as well as with other, less commonly found conditions.^2^^,^^3^ In summary, 61% of cutaneous horns originate from benign lesions, 23.2% from premalignant lesions, and 15.7% from malignancies.^4^ It can be difficult to differentiate between benign and malignant lesions; however, the latter appear to be more commonly associated with the male sex, older age, pain, photo-exposed areas, and large horn dimensions.^3^

Large cutaneous horns are rare and more commonly occur on a malignant base. Associations with immunodeficiency and, more specifically, oral GVHD have not yet been reported but seem plausible given the frequently encountered underlying viral mechanisms. Subsequent cancer manifesting in organs and tissues affected by chronic GVHD, however, is a well-known concern. Chronic graft-versus-host disease affects approximately one-half of patients who survive for 2 years after HSCT.^5^^,^^6^ Oral chronic GVHD has a reported prevalence ranging from 45% to 83% in patients who develop chronic GVHD.^7^

Traditionally, it has been stated that chronic GVHD begins more than 100 days post-HSCT. This definition was revised following the 2005 and 2014 publication of consensus papers from The National Institutes of Health, and currently, the diagnosis of chronic GVHD is made primarily based on clinical presentation.^8^ Oral chronic GVHD is based on at least one diagnostic manifestation (lichen planus-like features) or at least one distinctive manifestation (erythematous and ulcerative changes) with a pertinent biopsy, laboratory test, or other tests that support the diagnosis.^8^

In our patient, lichenoid lesions and oral white plaques were predominant. Oral white plaques as a diagnostic feature, as well as signs of dysplastic changes, have received little attention in the literature. A recent cohort study found that 50% of patients developing dysplasia of malignant oral changes in chronic GVHD had white plaques.^9^

Multiple studies have reported an elevated risk of nonmelanoma skin cancers, namely basal cell carcinoma and squamous cell carcinoma, after HSCT.^10^ One study focusing on moderate or severe chronic GVHD found that the 10-year cumulative incidence of nonmelanoma skin cancers was 15.5%, and that of all other skin cancer was 13.8%.^5^ Furthermore, long-term follow-up of these patients also suggests an increased risk for recurrence of oral carcinomas. Treatment of oral chronic GVHD consists of topical high-dose and ultrahigh-potency corticosteroids. In severe and refractive lesions, an intralesional steroid injection may be considered.^8^^,^^11^ Regular dermatological and dental evaluations are critical because of the risk for dental caries and secondary malignancies.^11^^,^^12^

Over the past decade, new interventions have been introduced for treating chronic GVHD; currently, sirolimus, rituximab, imatinib, ibrutinib, and ruxolitinib are more commonly used. Nonpharmacological modalities include extracorporeal photopheresis and phototherapy.^13^ Apart from regular histological examination of suspect lesions, there is no need for surgery in the management of oral chronic GVHD.

Timely diagnosis and treatment are recommended to prevent malignant transformation, if not yet present, and to reduce the psychosocial stress associated with these lesions. When surgical excision of cutaneous horns is attempted, adequate section margins (3-10 mm) are required due to the association with underlying malignancy, and subsequent histopathological evaluation is indispensable.^1^^,^^2^^,^^14^^,^^15^ Alternative treatments include electrocautery, cryotherapy, and laser surgery. However, these treatments are inferior because they do not result in an available specimen for histopathological examination. In our case, there was a high suspicion of malignancy due to the recurrent nature and the extent of the lesions, as well as the long-standing white mucosal aspect after classic chronic GVHD. A surgical shave with deep biopsies was performed as the treatment of choice. Regular follow-up occurred every 3 months thereafter.

Cutaneous horns can present as asymptomatic lesions and are benign in almost 2 out of 3 cases; however, the risk of underlying malignancy should not be underestimated. Standard treatment should consist of excision with 3-10 mm margins and histopathological examination, as lip shaves often leave the base of the lesion intact, leading to recurrence and inadequate pathologic examinations. To date, few cases could be found in the literature on cutaneous horns associated with GVHD, let alone their extended presence on the lower lip, as described in this case.

## Conflicts of interest

None disclosed.
